# Neonatal umbilical cord blood transplantation halts skeletal disease progression in the murine model of MPS-I

**DOI:** 10.1038/s41598-017-09958-9

**Published:** 2017-08-25

**Authors:** Isabella Azario, Alice Pievani, Federica Del Priore, Laura Antolini, Ludovica Santi, Alessandro Corsi, Lucia Cardinale, Kazuki Sawamoto, Francyne Kubaski, Bernhard Gentner, Maria Ester Bernardo, Maria Grazia Valsecchi, Mara Riminucci, Shunji Tomatsu, Alessandro Aiuti, Andrea Biondi, Marta Serafini

**Affiliations:** 1 Dulbecco Telethon Institute, Centro Ricerca M. Tettamanti, Department of Pediatrics, University of Milano-Bicocca, Monza, 20900 Italy; 2 Centro di Biostatistica per l’epidemiologia clinica, Department of Health Sciences, University of Milano-Bicocca, Monza, 20900 Italy; 3 Department of Molecular Medicine, Sapienza University, Rome, 00161 Italy; 4 Department of Biomedical Research, Alfred I. duPont Hospital for Children, Wilmington, DE 19803 USA; 5 Department of Biological Sciences, University of Delaware, Newark, DE 19716 USA; 6 San Raffaele Telethon Institute for Gene Therapy (SR-TIGET), San Raffaele Scientific Institute, Milan, 20132 Italy; 7 Vita Salute San Raffaele University, Milan, 20132 Italy; 8 Department of Pediatrics, University of Milano-Bicocca, Monza, 20900 Italy

## Abstract

Umbilical cord blood (UCB) is a promising source of stem cells to use in early haematopoietic stem cell transplantation (HSCT) approaches for several genetic diseases that can be diagnosed at birth. Mucopolysaccharidosis type I (MPS-I) is a progressive multi-system disorder caused by deficiency of lysosomal enzyme α-L-iduronidase, and patients treated with allogeneic HSCT at the onset have improved outcome, suggesting to administer such therapy as early as possible. Given that the best characterized MPS-I murine model is an immunocompetent mouse, we here developed a transplantation system based on murine UCB. With the final aim of testing the therapeutic efficacy of UCB in MPS-I mice transplanted at birth, we first defined the features of murine UCB cells and demonstrated that they are capable of multi-lineage haematopoietic repopulation of myeloablated adult mice similarly to bone marrow cells. We then assessed the effectiveness of murine UCB cells transplantation in busulfan-conditioned newborn MPS-I mice. Twenty weeks after treatment, iduronidase activity was increased in visceral organs of MPS-I animals, glycosaminoglycans storage was reduced, and skeletal phenotype was ameliorated. This study explores a potential therapy for MPS-I at a very early stage in life and represents a novel model to test UCB-based transplantation approaches for various diseases.

## Introduction

Haematopoietic stem cell transplantation (HSCT) can cure or greatly ameliorate a wide variety of genetic diseases, including defects of haematopoietic cell production or function and metabolic diseases mainly affecting solid organs^1^. In post-natal life, haematopoietic stem cells (HSCs) reside in the bone marrow (BM), so this was historically the first source of cells employed for HSCT. However, immediately after birth, HSCs can still be found in the fetal blood that flows in the umbilical cord vessels (umbilical cord blood, UCB). Unrelated donor UCB has several potential advantages over BM for HSCT, since it offers a relative ease of procurement, a greater degree of HLA (humal leukocyte antigen)-mismatch, with increased probability to find a suitable donor and lower incidence of acute and chronic graft versus host disease (GVHD), and reduced risk of viral infections (like Epstein-Barr virus and Cytomegalovirus)^1–4^. Furthermore, in the specific case of transplantation for inborn errors of metabolism (IEMs), UCB transplantation (UCBT) shows two significant extra-advantages over BM transplantation (BMT)^3, 5–7^. First, the availability of cells to transplant is more rapid, thanks to the augmented probability to find HLA-matched donors and the existence of cord blood banks where UCB units are stored frozen and ready to use. This factor is of primary importance because in many IEMs the timing of the treatment has a strong impact on patient outcome. Additionally, more patients transplanted with UCB achieve full donor chimerism and thus can obtain a normalization of the deficient enzyme levels in biological fluids, with consequent clinical benefits^3, 6, 8^.

New strategies and novel developments are expected to improve engraftment and reconstitution, and to enable *in utero* or neonatal UCB-based transplantation for early therapy of these diseases^2, 9^. Thus, convenient small animal models of these disorders are essential to investigate these developing strategies in the field of HSCT, including the use of alternative cellular sources and/or genetically modified HSCs.

Even though immunodeficient mouse models of many genetic disorders are available, in which the transplantation of human HSCs is feasible, many diseases lack an immunocompromised model that could fully recapitulate their clinical manifestations. In the case of Mucopolysaccharidosis type I-Hurler syndrome (MPS-IH), a lysosomal storage disease due to mutations in the α-L-iduronidase (*IDUA*) gene, immunocompetent mouse models have been deeply characterized for several features typical of this complicated disorder^10–13^. Recently, MPS-I immunocompromised models have been generated, but some aspects representative of the disease are not completely investigated yet^14, 15^. In this disorder, the absence of IDUA activity causes the progressive accumulation of glycosaminoglycans (GAGs) in tissues, which leads to multiple organ dysfunction, with central nervous system involvement and various skeletal anomalies known overall as dysostosis multiplex^16, 17^. The current first line therapy for MPS-IH is HSCT, which provides a constant reservoir of enzyme replacement through the engraftment of donor cells, and the use of UCB as stem cell source seems to guarantee the best results. However, transplantation is not completely effective in ameliorating bone abnormalities and neurocognitive dysfunctions, especially when it is performed late in childhood^7, 8, 18, 19^. Clinical and preclinical evidences attest that the precociousness of the treatment is critical to prevent long-term pathological consequences^20^. For this reason, we tested an UCBT approach at early age in MPS-I murine model^10^, to investigate a novel and promising therapeutic strategy. Very few data are present in the literature about murine UCBCs and their transplantation. Attempts to mimic UCBT have been made with either fetal liver cells, blood or BM collected from mouse fetuses during the last third of pregnancy, or newborn blood^21–27^, but, to our knowledge, no published data exists about the transplantation of murine UCB into newborn recipients, in particular in a mouse model of disease attesting a clinical correction.

Building upon the data from our previous study where we observed that the transplantion of normal BM into newborn MPS-I mice, soon after the placental protection, can prevent GAGs accumulation in multiple organs and the distinctive skeletal dysplasia^28^, in this study we provide an extensive description of murine UCB cells (UCBCs) features, in comparison with adult BM cells (BMCs). We characterized UCBCs *in vitro*, by flow cytometry and colony forming cell (CFC)-assay, and assessed the repopulating ability of UCBCs in conditioned adult and newborn wild-type (WT) mice. Finally, we focused on the pathological setting and investigated a novel treatment strategy based on the transplantation of UCBCs in MPS-I mice at birth. We extensively evaluated the outcome of this therapy, regarding restoration of enzyme activity, reduction of GAG deposits in plasma and visceral organs, and correction of the skeletal phenotype.

## Results

### Collection of UCBCs and their comparison with adult BMCs *in vitro*

**Figure 1:**
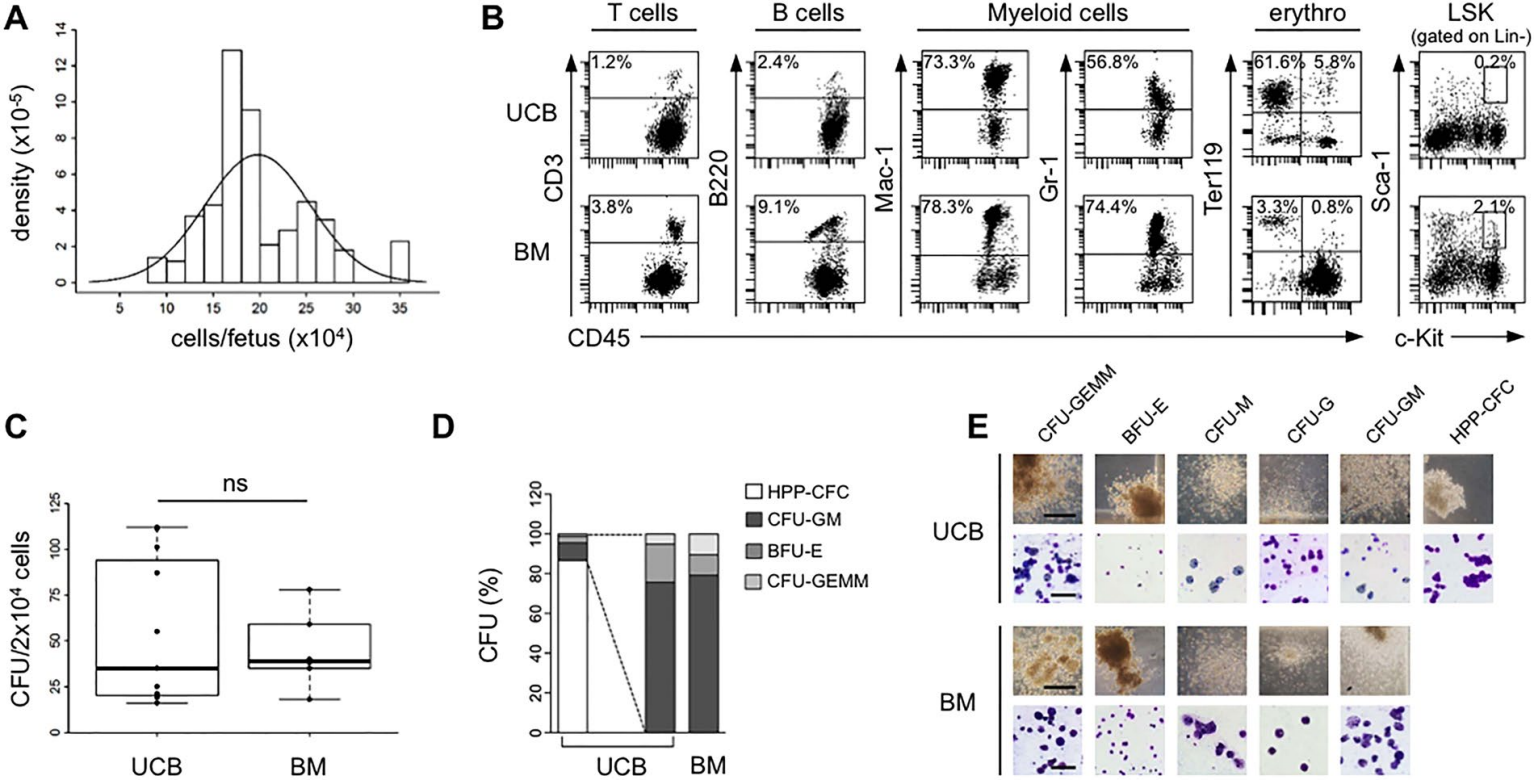
Murine UCBCs have unique features compared with BMCs. (**A**) Number of UCBCs obtained at day E18 from each fetus (n = 502 fetuses). The distribution of the medium number of cells per fetus was represented by density histogram with Gaussian approximation. (**B**) Representative flow cytometry analysis of UCB and BM haematopoietic subpopulations: T cells (CD45^+^CD3^+^), B cells (CD45^+^B220^+^), myeloid cells (CD45^+^Mac-1^+^ and CD45^+^Gr-1^+^), erythroid cells (TER-119^+^), and LSK cells (lin^-^Sca-1^+^c-Kit^+^). Percentages of lymphocytes and myeloid cells were referred to CD45^+^ leukocytes, percentage of Ter119^+^ was referred to all cells (after hypertonic treatment), and percentage of LSK cells was referred to Lin^-^ leukocytes. (**C**) Absolute number of haematopoietic colonies detected on methylcellulose at day 14 after plating 2 × 10^4^ UCB or BM cells/petri (n = 11 UCB, n = 6 BM). Data are represented by boxplot graphs, showing the exact data values by black dots. P = 1 with 2-sided Wilcoxon unpaired test. (**D**) Barplot with percentage of the different subtypes of HPP-CFC, CFU-GEMM, BFU-E and CFU-GM (CFU-M, CFU-G, and CFU-GM) among the total number of colonies obtained from UCB or BM. (**E**) Representative photographs of the different subtypes of haematopoietic colonies in UCB and BM (10X magnification, bar: 400 μm) and of their cytospin preparations stained with May-Grumwald Giemsa (200X magnification, bar: 200 μm). CFU-GEMM = Colony-Forming Unit-Granulocyte, Erythroid, Macrophage, Megakaryocyte; BFU-E = Burst-Forming Unit-Erythroid; CFU-M = Colony-Forming Unit-Macrophage; CFU-G = Colony-Forming Unit-Granulocyte; CFU-GM = Colony-Forming Unit-Granulocyte, Macrophage; HPP-CFC = High Proliferative Potential-Colony-Forming Cell.

We collected UCBCs at gestational day E18 from C57BL/6 pregnant dams, which carried a mean number of fetuses/dam of 6.97 (standard deviation [SD] 1.81; n = 72 dams). The mean number of UCBCs collected from each dam was 1.38 × 10^6^ (SD 4.51 × 10^5^), and it varied proportionally with the number of fetuses/dam (data not shown). The mean number of cells obtained from each fetus was 19.8 × 10^4^ (SD 5.64 × 10^4^) (Fig. 1A). Haematopoietic cells belonging to different lymphoid and myeloid lineages were found both in UCB and adult BM, as shown in the representative flow cytometry panels in Fig. 1B. However, the proportion of lymphocytes (T cells and B cells) and of myeloid cells (monocytes/macrophages and granulocytes) was higher in BM than in UCB, suggesting that UCB could contain less mature cell populations (Supplementary Table S1). Interestingly, Ter119^+^ erythrocytes were very few in BM after lysis but remained at a high percentage in UCB, probably because they are mostly immature and resistant to hypotonic shock (Supplementary Figure S2). Regarding the HSCs subset easily detectable within adult BMCs by Lin^−^Sca-1^+^c-kit^+^ (LSK) staining, in UCBCs specimens there was a reduced proportion of LSK cells (Fig. 1B). In the colony-forming cell (CFC) assay, performed to investigate the functionality of the haematopoietic progenitors, a similar frequency of colonies was found in UCB and BM (median 35.0 colonies/plate in UCB, range from 16 to 112, and 39.0 colonies/plate in BM, range from 18 to 78; p = 1) (Fig. 1C). However, in UCB the majority of the colonies (93.2%) had a peculiar morphology, consisting of colonies containing large blast-like cells on a single layer (Fig. 1D,E). These cells resemble the previously defined High Proliferative Potential-Colony-Forming Cells (HPP-CFC), primitive multipotent progenitor cells absent in BM-derived colonies^23^. Excluding HPP-CFCs, the relative distribution of the other colony subtypes (CFU-GEMM, BFU-E, CFU-GM) did not differ between UCB and BM (p = 0.06, Chi-square test) (Fig. 1D). The different subtypes of UCB and BM haematopoietic colonies were morphologically indistinguishable (Fig. 1E).

### UCB contains long-term multi-lineage repopulating haematopoietic stem cells

**Figure 2:**
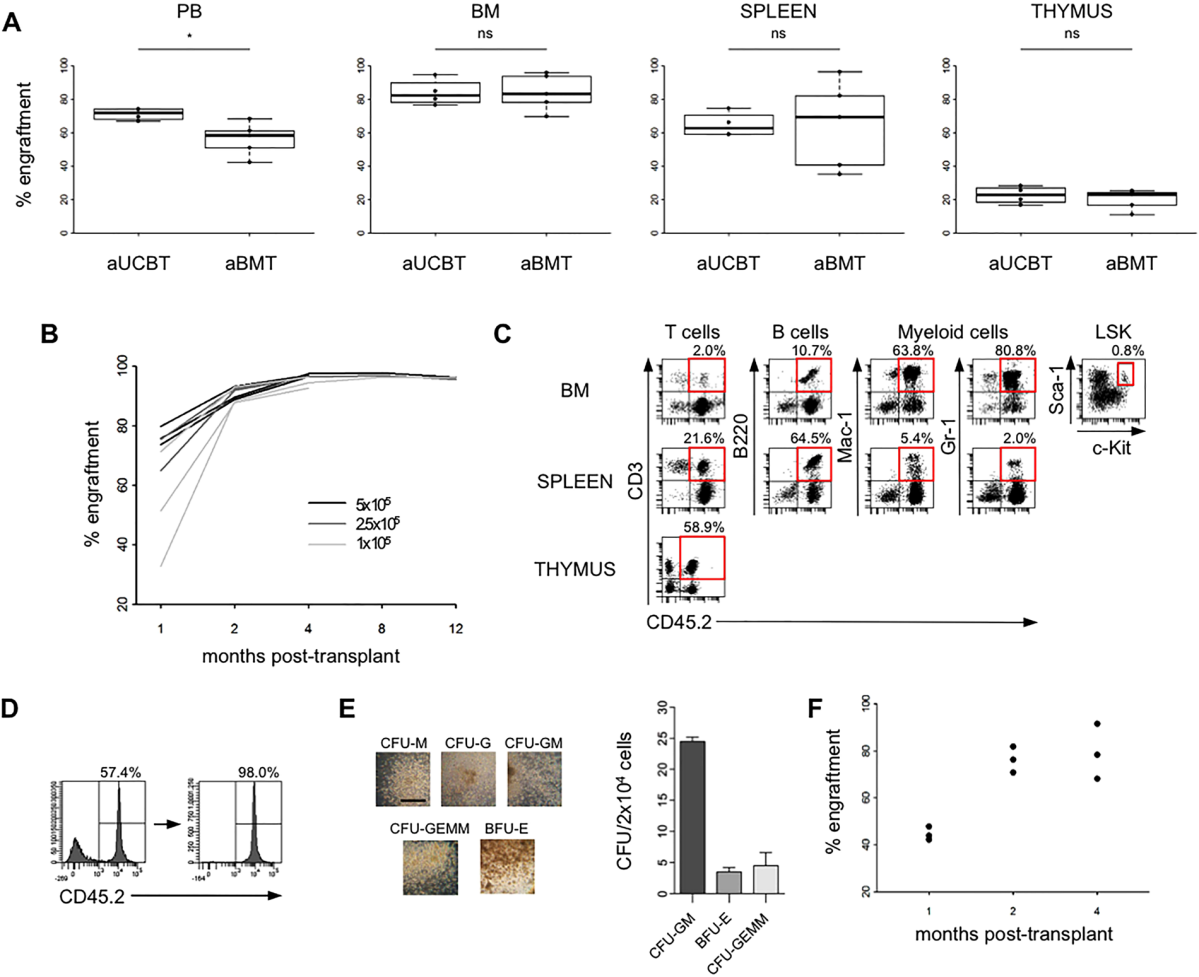
Murine UCBCs demonstrate long-term multi-lineage haematopoietic repopulating activity in adult transplantation setting. (**A**) Levels of donor chimerism [donor CD45 cells/(donor + host CD45 cells) × 100] were determined by flow cytometry in the haematopoietic organs of adult lethally-irradiated recipients at 1 month after the transplantation of 5 × 10^5^ UCBCs (aUCBT) or BMCs (aBMT) (n = 4 aUCBT, n = 5 aBMT). *p ≤ 0.05 by Wilcoxon test. (**B**) Levels of chimerism analyzed serially in the PB of recipient mice between 1 and 12 months after the transplantation of 5 × 10^5^, 2.5 × 10^5^, or 1 × 10^5^ UCBCs/mouse (each line in the plot represents a single mouse). (**C**) Representative lineage distribution of UCB-derived cells in the BM, spleen, and thymus of recipient mice at 4 months after aUCBT. Dot plots to determine donor-derived T cells (CD45.2^+^CD3^+^), B cells (CD45.2^+^B220^+^), myeloid cells (CD45.2^+^Mac-1^+^ and CD45.2^+^Gr-1^+^), and LSK cells (CD45.2^+^lineage^-^Sca-1^+^c-Kit^+^) are shown. (**D**) FACS sorting of CD45.2^+^ UCB-derived cells from the BM of a primary aUCBT recipient at 4 months after transplantation. (**E**) Representative photographs and count of the different subtypes of haematopoietic colonies on methylcellulose formed by UCB-derived (CD45.2^+^) BM sorted cells (10X magnification, bar: 400 μm). (**F**) Donor chimerism in the PB of secondary mice after the transplantation of 3 × 10^6^ UCB-derived (CD45.2^+^) BM sorted cells (n = 3 recipient mice). Each black dot in the plot represents a single mouse, analyzed at 1, 2, and 4 months after transplant.

Before performing the transplantion of UCBCs into newborn MPS-I mice, we assessed whether they were able to rescue lethally-conditioned adult mice, to differentiate into cells of lymphoid and myeloid lineages, to persist long-term, and to repopulate in serial transplants. In an initial set of experiments, lethally-irradiated adult C57BL/6-CD45.1 mice were transplanted with 5 × 10^5^ CD45.2^+^ UCBCs (adult UCBT group, aUCBT) or with the same number of murine adult BMCs (adult BMT group, aBMT). At 1 month after transplantation, the short-term engraftment of donor cells was assayed in peripheral blood (PB), BM, spleen, and thymus of the recipients, by flow cytometric analysis of the leukocytes marked with anti-CD45.1 and anti-CD45.2 antibodies (Fig. 2A). In the aUCBT group, median engraftment in PB was 71.7% (range from 66.9% to 74.3%), even higher than the one in the aBMT group that was 58.4% (range from 42.3% to 68.3%; p = 0.03). In the BM, the median donor chimerism was 82.5% in the aUCBT group and 83.3% in the aBMT group, while it was, respectively, 62.7% and 69.3% in the spleen, and 22.8% and 23.2% in the thymus, without significant differences between UCBCs and BMCs (p = 1, p = 0.90, p = 0.56, respectively). We next assessed if it was possible to use lower doses of UCBCs (2.5 × 10^5^ and 1 × 10^5^ cells/mouse) to establish long-term, stable chimerism. Rates of engraftment (number of surviving mice with ≥1% donor cells/total number of transplanted mice) were 100% for all the tested doses. While at 1 month after transplantation the level of donor cell engraftment in PB depended on the transplanted cell dose, beginning from 4 months after transplantation the engraftment reached values over 90% in all the experimental groups (Fig. 2B). More importantly, UCBCs showed long-term repopulation ability, since PB engraftment was maintained up to 12 months after aUCBT. The engraftment in other haematopoietic organs was assayed at 4 months after transplantation, and it reached a median of 98.2% in BM, 93.5% in spleen, and 92.1% in thymus (data not shown). The presence of cells arisen from the original UCBCs (CD45.2^+^ cells) in both lymphoid (T and B cells) and myeloid (monocytes/macrophages and granulocytes) lineages was attested in BM, spleen and thymus by flow cytometry (Fig. 2C). CD45.2^+^ LSK cells were also found in the BM of recipient mice, suggesting that, even if LSK cells were detected as a very rare population in UCB, UCBCs were able to repopulate also the HSCs pool in the recipients’ BM (Fig. 2C). To evaluate the functionality of UCB-derived haematopoietic progenitors, CD45.2^+^ cells were sorted 4 months after aUCBT from the BM of recipients and were tested in a CFC-assay, showing the differentiation in colonies belonging to all the different subtypes (Fig. 2D,E). Finally, a secondary transplantation assay into lethally-irradiated CD45.1 recipients was performed, to verify whether UCBCs contained long-term HSCs. The presence of sustained and durable levels of PB engraftment in secondary mice confirmed self-renewal and long-term repopulation capability of UCB-derived HSCs (Fig. 2F). CD45.2^+^ mature subpopulations and LSK cells were also present in BM of secondary mice (data not shown).

### Transplantation of UCBCs in the neonatal setting

**Figure 3:**
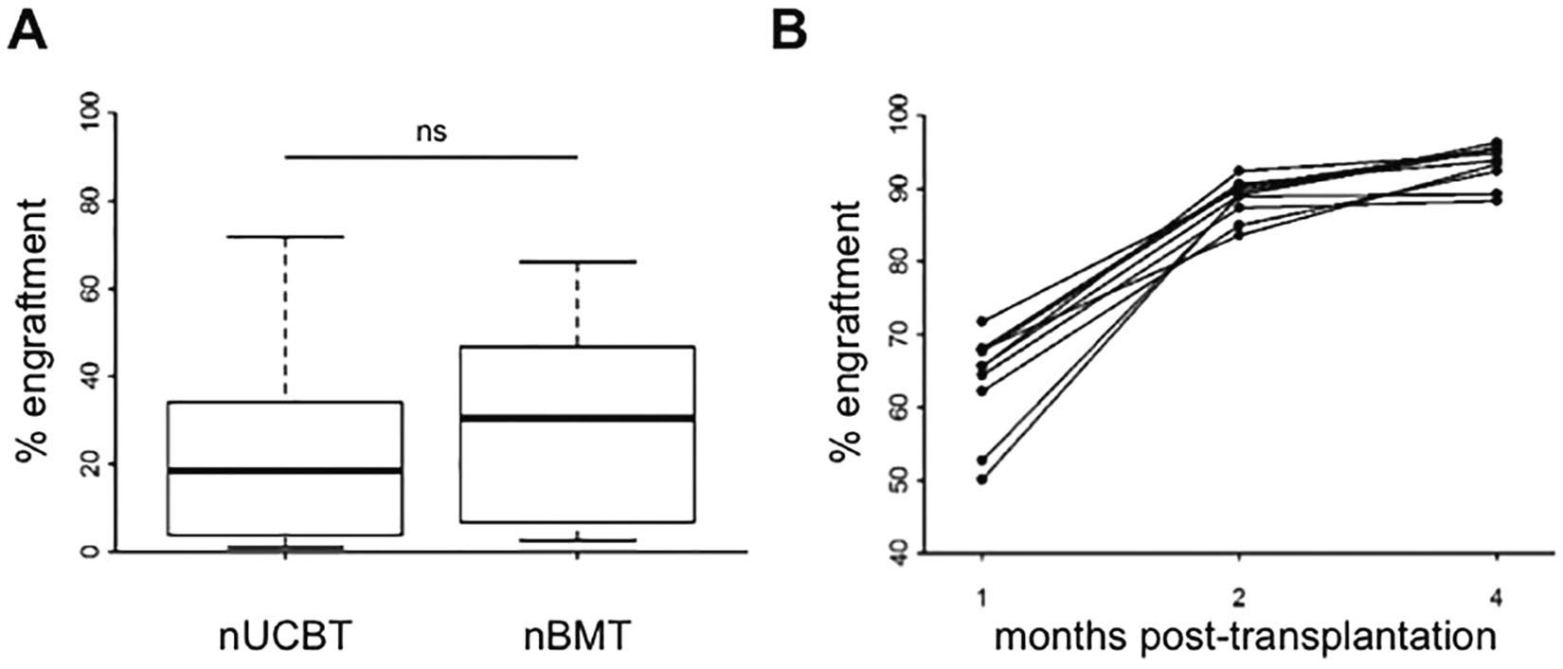
Murine UCBCs confirm long-term multi-lineage haematopoietic repopulating activity in neonatal transplantation setting. (**A**) Levels of donor chimerism were determined by flow cytometry in the PB of busulfan-conditioned newborn mice at 1 month following the transplantation of 2 × 10^5^ UCBCs (nUCBT) or BMCs (nBMT) cells (n = 68 nUCBT, n = 28 nBMT; p = 0.10 by Wilcoxon test). (**B**) Serial analysis of donor chimerism in the PB of nUCBT recipient mice performed at 1, 2, and 4 months after transplant (n = 10, each line in the graph represents a single mouse).

For the transplantation of newborn mice, we adopted a previously established protocol with few modifications^28^. After conditioning with busulfan, CD45.2 newborn mice were intravenously transplanted with either CD45.1^+^ UCBCs (neonatal UCBT group, nUCBT) or adult BMCs (neonatal BMT group, nBMT). The cell dose we defined to transplant (2 × 10^5^ cells/mouse) was ideally comparable with the mean number of UCBCs harvested from a single fetus. At 1 month after transplantation, no difference in the PB engraftment was observed between nUCBT group (median: 18.5%, range from 1.0% to 71.8%) and nBMT group (median: 30.5%, range from 2.6% to 66.1%; p = 0.10, including in our analysis only successfully transplanted mice with donor chimerism ≥1%) (Fig. 3A). nUCBT mice with PB engraftment ≥50% at 1 month were analyzed serially at 2 and 4 months, and the levels of engraftment in PB increased over time, approaching full donor chimerism (Fig. 3B). The engraftment in BM, spleen, and thymus, the differentiation of transplanted UCBCs in lymphoid and myeloid lineages, and the retention of LSK cells were evaluated at 6 months post transplantation, and the results were comparable with the ones obtained in aUCBT mice (Supplementary Figure S1 and data not shown). Thus, we could also confirm in the neonatal setting the haematopoietic repopulation ability of UCBCs.

### Engraftment and biochemical features of MPS-I mice receiving nUCBT

**Figure 4:**
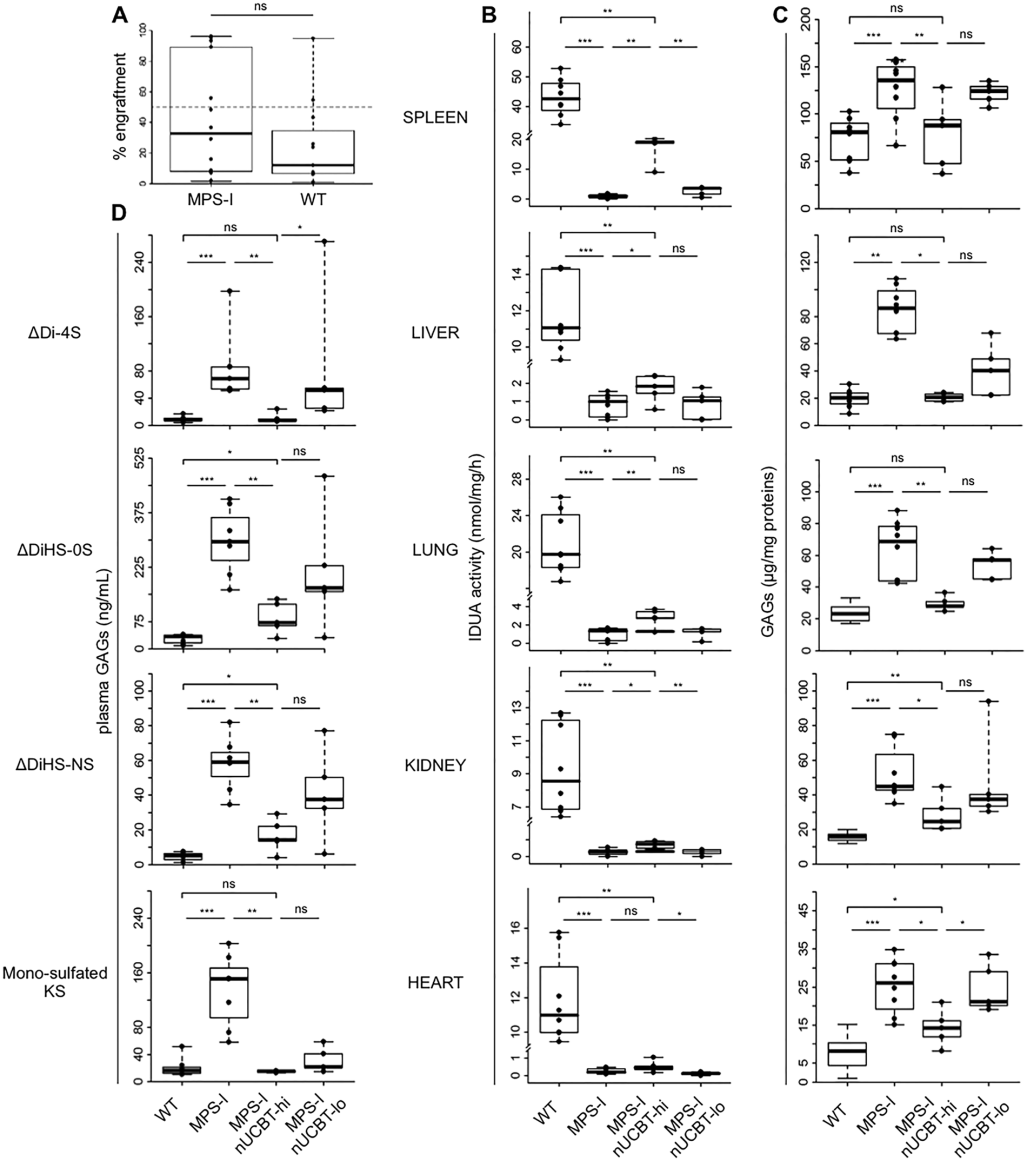
Neonatal UCBT prevents GAGs accumulation in MPS-I mice. (**A**) Donor chimerism (percentage of CD45.1^+^ cells) determined by flow cytometry in the PB of recipient MPS-I and WT mice at 20 weeks (time of sacrifice) after nUCBT (n = 12 for MPS-I, n = 12 for WT; p = 0.24 by Wilcoxon test). Dashed line indicates the level of 50% donor engraftment, and identifies the highly-engrafted mice group (with ≥50% donor cells in PB, nUCBT-hi). (**B**) IDUA activity in spleen, liver, lung, kidney, and heart of WT (n = 8), MPS-I (n = 8), MPS-I nUCBT-hi (n = 5), and MPS-I nUCBT-lo mice (n = 5). (**C**) GAG levels in the indicated organs of the same WT, MPS-I, MPS-I nUCBT-hi, and MPS-I nUCBT-lo mice. (**D**) Levels of ΔDiHS-0S, ΔDiHS-NS, ΔDi-4S, and mono-sulfated KS in the plasma of the mice. *p ≤ 0.05, **p ≤ 0.01, ***p ≤ 0.001 by Wilcoxon test.

To investigate whether nUCBT could represent a curative treatment for metabolic diseases, we applied the settled protocol to the MPS-I mouse model. Newborn MPS-I and WT mice were transplanted with healthy UCBCs and evaluated at 20 weeks of age for their PB engraftment, IDUA activity in organs, GAGs accumulation in organs and plasma, and skeletal phenotype. PB engraftment did not differ between MPS-I and WT mice at the time of sacrifice (median 38.7% in MPS-I, range from 1.6% to 96.2%; 9.8% in WT, range from 1.0% to 94.8%; p = 0.24) (Fig. 4A). Among MPS-I nUCBT mice, 5 of 12 mice presented a high haematopoietic chimerism, defined as more than 50% donor CD45.1^+^ cells in PB at 20 weeks after nUCBT (median engraftment: 93.3%, range from 55.7% to 96.2%). Hence, we included this subgroup of highly engrafted mice (named MPS-I nUCBT-hi) in all the studies reported hereafter. Five of 12 mice, instead, had a low haematopoietic chimerism, defined as less than 10% donor CD45.1^+^ cells in PB at 20 weeks after nUCBT, and were grouped as low engrafted mice (MPS-I nUCBT-lo). IDUA activity was evaluated in the spleen, liver, lung, kidney, and heart of MPS-I nUCBT mice compared with age-matched untreated WT and MPS-I mice. IDUA activity, which is absent in MPS-I mice and not restored in MPS-I nUCBT-lo mice, was partially increased in MPS-I nUCBT-hi mice in all the tissues analyzed, particularly in spleen, where the average values in MPS-I nUCBT-hi mice reached 40% of average WT values (Fig. 4B). In the same harvested tissues, we quantified GAG levels, showing that MPS-I nUCBT-hi animals displayed a statistically significant reduction in GAGs storage material in all organs, in comparison with untreated MPS-I mice (p ≤ 0.03 for all organs) (Fig. 4C). The average reduction on MPS-I is over 50% for spleen, heart, lung and kidney. In particular, in spleen, liver, and lung of MPS-I nUCBThi animals GAG levels completely normalized (MPS-I nUCBThi *vs*. WT, p = 0.76, p = 1, and p = 0.11, respectively) (Fig. 4C). To further confirm the occurred correction, we measured the levels of plasma GAGs (HS-0S, HS-NS, DS, and mono-sulfated KS), showing that the levels of these GAGs were significantly reduced in MPS-I nUCBT-hi compared to untreated MPS-I mice (Fig. 4D). Of note, in MPS-I nUCBT-hi mice the level of mono-sulfated KS, which has been associated with severity of skeletal dysplasia in the mouse model of MPS-I, was similar to WT animals (MPS-I nUCBThi *vs*. WT; p = 0.72) (Fig. 4D). Instead, GAG levels in both peripheral organs and plasma were not consistently reduced in MPS-I nUCBT-lo mice (Fig. 4C,D). Taken together, these biochemical data prove that nUCBT greatly corrects the error of metabolism in these tissues in animals with high donor chimerism.

### Prevention of dysostosis in MPS-I mice receiving nUCBT

**Figure 5:**
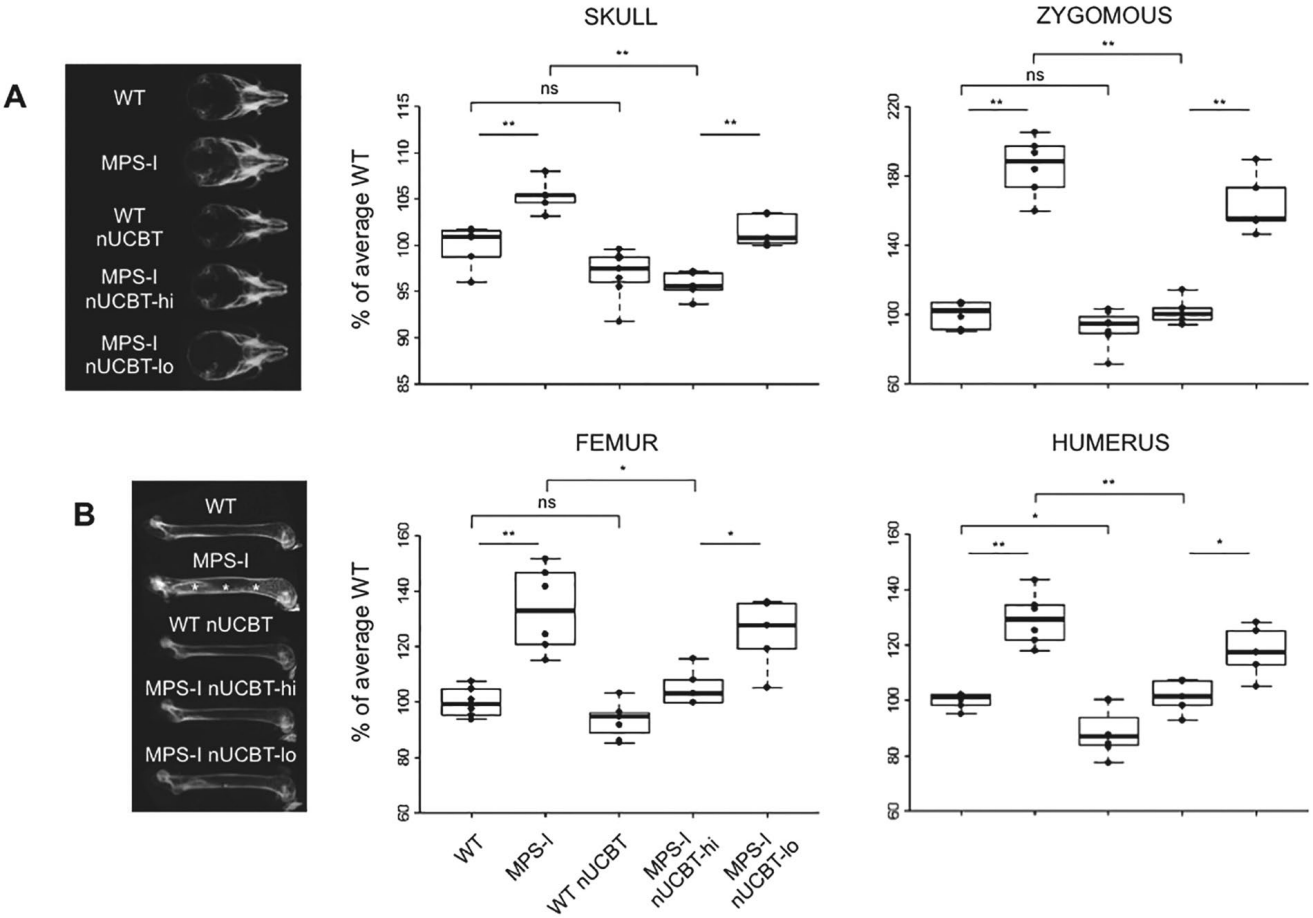
Neonatal UCBT prevents bone thickening in MPS-I mice. (**A**) On the left, representative radiographs of the skull of 20-weeks-old WT, MPS-I, WT nUCBT, MPS-I nUCBT-hi, and MPS-I nUCBT-lo mice. On the right, measurements of the skull width and zygomous width, performed on radiographs of WT (n = 6, 3 males and 3 females), MPS-I (n = 6, 3 males and 3 females), WT nUCBT (n = 7, 3 males and 4 females), MPS-I nUCBT-hi (n = 5, 3 males and 2 females), and MPS-I nUCBT-lo mice (n = 5, 3 males and 2 females). (**B**) On the left, representative radiographs of the femur of 20-weeks-old WT, MPS-I, WT nUCBT, MPS-I nUCBT-hi, and MPS-I nUCBT-lo mice. The increase in meta-diaphyseal bone density observed in MPS-I (asterisks) is significantly prevented in MPS-I nUCBT-hi mice. On the right, measurements of the femur and humerus widths, performed on the radiographs of the same animals as in panel A. *p ≤ 0.05, **p ≤ 0.01, by Wilcoxon test.

Dysostosis multiplex is the well-known skeletal consequence of MPS-I in humans and mouse models. In particular, the MPS-I model adopted in this work shows abnormal craniofacial bone morphology and progressive thickening of the long bone segments. At the age of sacrifice (20 weeks) radiographic analyses confirmed a marked increase in the width of the skull and of the zygomatic arches in untreated MPS-I mice compared to WT animals (Fig. 5A). Instead, in MPS-I nUCBT-hi mice, a significant reduction of these parameters was observed (skull width p = 0.008; zygomatic arch width p = 0.005, MPS-I nUCBT-hi *vs*. untreated MPS-I mice).

A similar trend was observed in the femur and humerus, where the thickening and the meta-diaphyseal sclerosis of the skeletal bones of MPS-I mice revealed by radiographic analysis were prevented in MPS-I nUCBT-hi animals (Fig. 5B).

Considering that busulfan toxicity *per se* could cause a reduction in bone dimensions of treated mice regardless of their genotype, we adopted a regression model capable of separating the adverse effect of busulfan treatment from the therapeutic effect of transplantation on MPS-I^28^. By this analysis, we obtained the confirmation of the differential effect of the treatment on MPS-I attributable to transplantation only (Supplementary Table S2). Moreover, considering the MPS-I nUCBT-lo group, the improvement in radiographic measurements was limited compared to MPS-I nUCBT-hi, confirming the importance of high donor chimerism for disease correction (Fig. 5A and B).

**Figure 6:**
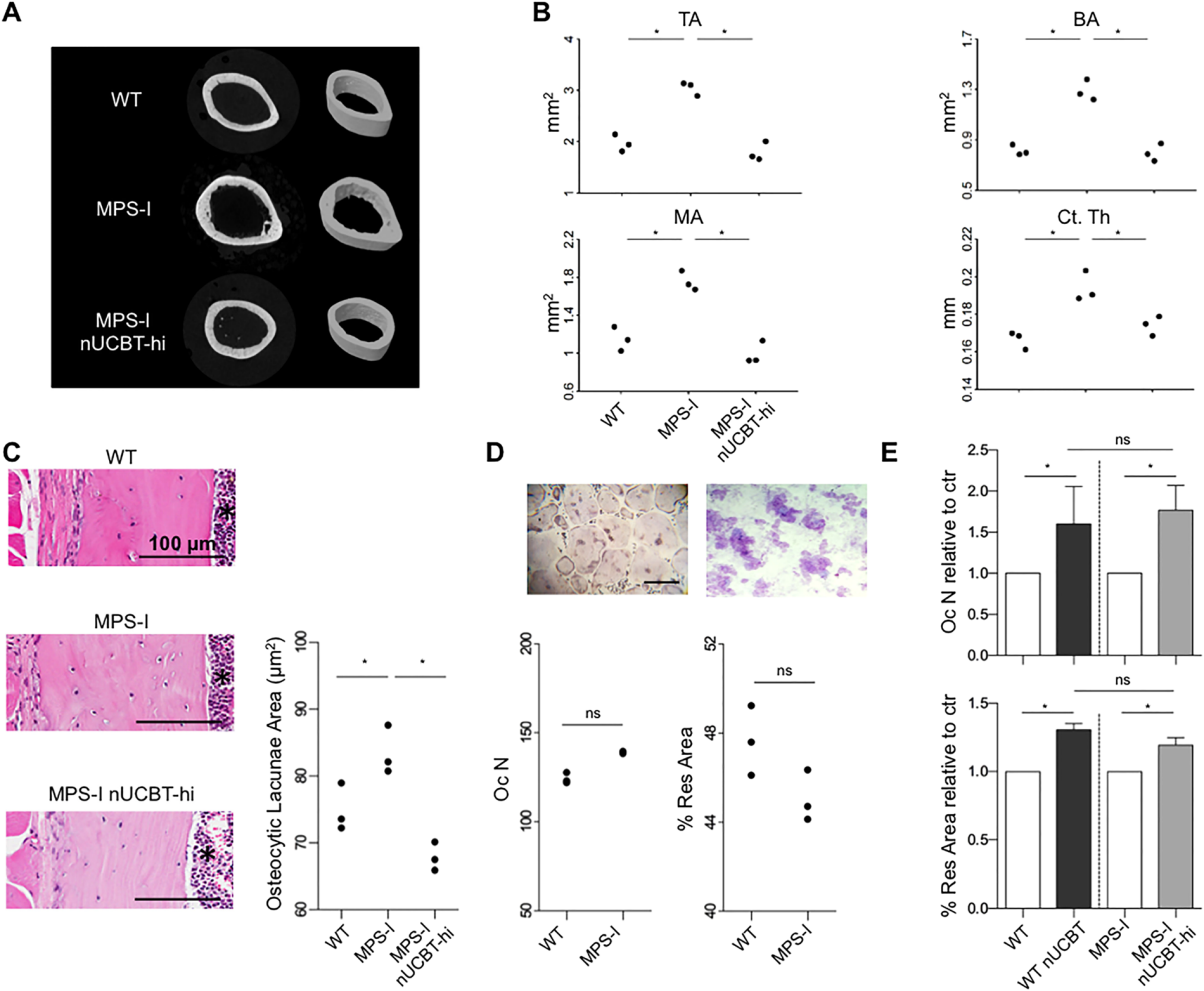
Neonatal UCBT improves cortical bone architecture in MPS-I mice. (**A**) Representative 2D and 3D micro-CT images showing regions of femoral cortical bone in WT, MPS-I, and MPS-I nUCBT-hi 20-weeks-old male mice. (**B**) Graphs representing the measurement of total area (TA/mm2), bone area (BA/mm2), medullary area (MA/mm2), and cortical thickness (Ct.Th/mm) of 3 mice per group (WT, MPS-I, and MPS-I nUCBT-hi). (**C**) Representative haematoxylin and eosin stained histological sections of the femur cortical bone at the mid-diaphysis are shown in the panels on the left. The graph illustrates the measurement (mean ± SD) of the area of the osteocytic lacunae within the femur cortical bone of 3 mice per group (WT, MPS-I, and MPS-I nUCBT-hi). The BM cavity is indicated by an asterisk. Bar: 100 μm. (**D**) Representative pictures of TRAP-positive multinucleated osteoclasts differentiated *ex vivo* (on the left, magnification 10X; bar: 300 μm) and their resorption plots on dentin slices (on the right, magnification 20X). Quantification of number and resorptive capacity of osteoclasts obtained by *ex vivo* differentiation of BM cells arisen from untreated WT and MPS-I mice (n = 3 male mice per group). (**E**) Fold increase of the number and resorptive capacity of the osteoclasts obtained from treated mice, relative to control (untreated mice of the respective genotype) (mean ± SD). *p ≤ 0.05 by Wilcoxon test.

Micro-computed tomography (micro-CT) scans and histomorphometry performed on the femurs of male mice again highlighted the improvement of the skeletal phenotype in the MPS-I nUCBT-hi group. 2- and 3D micro-CT images revealed that the endocortical perimeter of MPS-I femurs appeared distinctly irregular at 20 weeks and returned to normal in MPS-I nUCBT-hi mice (Fig. 6A). Specifically, all the examined parameters (total cortical area, cortical bone area, medullary area, and cortical thickness) were ameliorated in MPS-I nUCBT-hi mice, demonstrating the impact of the high donor engraftment on femoral architecture (Fig. 6B). In addition, comparative histomorphometric analysis of the femur cortical thickness at mid-diaphysis and the area of the osteocytic lacunae confirmed the benefit of nUCBT on bone abnormalities in MPS-I mice (Fig. 6C and data not shown).

Considering the impact that osteoclastogenesis seems to have on MPS-I disease^29^, we determined the effect of nUCBT on osteoclast numbers and function. There were no significant differences in the ability of BM cells derived from untreated WT and MPS-I mice to differentiate into TRAP-positive multinucleated osteoclasts *ex vivo* and in their resorptive capacity *in vitro* when cultured on dentine slides (Fig. 6D). Nonetheless, nUCBT treatment caused increased osteoclastogenesis regardless of the mice’s genotype (p ≤ 0.05, treated *vs*. untreated mice) (Fig. 6E), although an effective reduction of bone mineral density could not be found *in vivo* (Supplementary Figure S3).

## Discussion

UCB is a clinically useful reservoir of HSCs and progenitor cells for the treatment of a wide variety of genetic diseases, particularly attractive for transplantation of infants and small children.

To fully realize the therapeutic potential of UCBT early after birth, it is fundamental to develop novel tools to test its efficacy in different defects. MPS-I offers an ideal model, since the relevance of UCBT in the treatment of this condition is well-known in clinic.

In this study, we demonstrate that the transplantation of murine UCBCs into lethally-irradiated congenic recipients long-term reconstitutes all blood cell lineages. Moreover, the BM of recipients contains cells capable of reconstituting the haematopoietic system of secondary hosts.

Furthermore, in the neonatal setting, MPS-I mice transplanted with UCBCs show high levels of chimerism with the donor healthy cells, that are both well tolerated and therapeutic. Indeed, the long-term engraftment results in the partial restoration of IDUA enzyme activity, clearance of GAGs storage, and significant improvement in altered bone architecture, with prevention of the skeletal phenotype.

In contrast to adult murine BM, the features of murine UCB have been poorly investigated. In a few studies, blood from late fetal and newborn mice has been employed, due to the similar hallmarks with UCB obtained at birth in human beings. In the current study, we used UCB collected from murine fetuses at embryonic day 18. Even if UCB contained few nucleated cells, the collected cell population comprised the most representative committed lineages (T cells, B cells, and myeloid cells), although in different proportions if compared to BMCs. Notably, the majority of T cells are immature, with a double positive CD4^+^CD8^+^ phenotype and low levels of TCRα/β, as similarly reported for human UCB^27, 30^. Both the low percentage of mature T cells and the weak reactivity of the numerous immature T cells can be responsible for the reduced incidence of GVHD in patients transplanted with UCB^31^. The almost complete absence of mature lymphocytes and the reduced number of innate immunity cells are allowed by the intra-uterine protection during fetal life^32^. Differently from murine adult BM, murine UCB is characterized by the presence of Ter119^+^ immature red blood cells resistent to hypotonic shock, including a population of nucleated red blood cells. These data are consistent with similar results reported for human UCB, which contains two distinct red cells populations, a minority of rapidly lysed cells and a majority of slowly disrupted cells^33^.

Regarding the HSCs subset, the proportion of LSK cells, easily detectable within adult BM, was very low in UCB. This is consistent with the findings of Migishima *et al*., who stated that murine UCB virtually lacked cells with the LSK phenotype representative of adult BM-derived HSCs^24^. Considering that UCB cells successfully reconstituted lethally irradiated recipients, the authors conclude that some phenotypic differences between BM and UCB HSCs may exist. A possibility is that UCB HSCs do not express Sca-1, since they found a population of Lin^−^c-Kit^+^ cells among the side population. Another possibility is that UCB HSCs express Mac-1 similarly to fetal liver HSCs, and consequently a LSK phenotype can be observed only if the anti-Mac-1 antibody is removed from the anti-lineage cocktail^34, 35^.

Furthermore, the majority of the colony progenitors was constituted by multipotent precursors that give rise to colonies with a peculiar blast-like morphology when cultured *in vitro*, resembling the previously-defined HPP-CFC^23^. As already reported, this population of haematopoietic cells demonstrating HPP-CFC activity begins to be present in the yolk sac and in the embryo and represents the earliest multi-potential precursors within the haematopoietic hierarchy than can be cultured without stromal support^36^. Similarly, human UCB cultures contain a higher proportion of immature, late developing, multi-potential colony-forming cells than adult BM cultures^37^.

Even though these findings indicate that UCB has a different composition compared to BM, UCBCs can engraft with an extent similar to adult BM. In the congenic context, we do not observe any post-transplantation delay in haematopoietic recovery, differently than previously reported by Li *et al*. in an allogeneic UCBT model^26^. Notably, the persistence of donor-derived lymphoid and myeloid lineages over 4 month after transplantation demonstrates the long-term function of the HSCs contained in UCB, considering that most precursors and short-term HSCs that repopulate soon after transplantation are short-lived and disappear within 3 to 4 months after transplant in mice^38^. It has been further demonstrated that T and B cells derived from UCB-HSCs are fully competent in immunological terms^25^. Long-term repopulating function of HSCs in UCB was definitively confirmed by the robust contribution to multi-lineage engraftment in secondary irradiated recipients. Thus, HSCs from late fetal blood have a long-term multi-lineage repopulating ability similar to those in adult BM, in agreement with the similar competitive repopulation capacity previously demonstrated by Harrison *et al*.^22^.

Using a myelo-ablative conditioning regimen based on busulfan described in our previous work^28^, we could demonstrate that also in the neonatal setting UCB has been able to repopulate the haematopoietic tissues, showing long-term multi-lineage reconstitution in mice transplanted at birth. Moreover, we showed that the number of cells derived from a single UCB sample can provide sufficient long-term repopulating ability to fully maintain a newborn recipient for at least 20 weeks.

To our knowledge, these are the first *in vivo* experiments carried out using UCBCs to perform a transplant at neonatal age. This new model of UCBT offers a potential tool to elucidate the biological features of the perinatal haematopoietic stem/progenitor cells and to develop early UCB-based therapies.

Notably, allogeneic murine late fetal or newborn blood has been transplanted in adult mouse models for prevention or treatment of autoimmune diseases such as type I diabetes and systemic lupus erythematosus^39, 40^, but never in models of genetic disorders at birth.

In Hurler disease, UCB has become in the most recent years the preferential stem cell source for affected infants and children because, in comparison with BM, this source demonstrated more immediate availability, higher donor chimerism, better enzyme recovery in blood, and superior engrafted-and-alive rates^8^. In our study, we provide evidence that neonatal UCBT in MPS-I mice allows efficient and long-term haematopoietic engraftment. Twenty weeks after neonatal UCBT, MPS-I mice with more than 50% replacement by donor-derived haematopoiesis demonstrated near-complete normal values of biochemical parameters in visceral organs as compared with affected control mice. Indeed, the level of GAGs, which is an indicator of the disease progression, in the majority of the tissues investigated was completely normalized, confirming the efficacy of an early approach based on the infusion of UCBCs. Notably, the keratan sulfate (KS) level, which could be considered a biomarker of skeletal dysplasia in MPSs^41, 42^, was normalized after neonatal UCBT in MPS-I mice. We then focused our studies on skeletal disease, considering that it is one of the unmet clinical needs of utmost importance in transplanted MPS-I patients.

Definitely, the reconstitution of normal haematopoiesis in MPS-I mice was associated with a consistent amelioration of bone pathology, as revealed by radiographic skeletal examination. Micro-CT scans and histomorphometry remarked the impact of the high donor engraftment on the internal architecture of the femurs of transplanted mice. This could be due to enzyme delivery by haematopoietic cells close to the bone and also to tissue reconstitution by other donor-derived multipotent stem cells. Indeed, we recently demonstrated that a rare population of cells within the non-haematopoietic fraction of UCB, named cord blood-borne fibroblasts, shows *in vitro* and *in vivo* chondrogenic ability and the specific capacity of generating *in vivo* bone and a BM stroma that supports functional haematopoiesis^43, 44^. Furthermore, Uchida *et al*. demonstrated that murine UCB transplantation could fully reconstruct not only haematopoietic cells, but also mesenchymal cell lineages able to differentiate into osteoblastic cells in response to environmental specific cues^45, 46^.

Using a statistical model that separates the therapeutic effects of UCBT on MPS-I bones from the toxic effect of busulfan treatment on bones of transplanted MPS-I or WT mice^28^, we could definitively demonstrate that neonatal UCBT reduced bone thickening in the skull, zygomatic arches, and long bone segments.

Another reported side-effect of the conditioning regimens with cyto-reductive chemotherapy agent such as busulfan is bone loss due to increased bone resorption^47^. In this sense, we observed a significant increase in the capacity of BM cells obtained from transplanted MPS-I mice to differentiate in TRAP-positive multinucleated osteoclasts *ex vivo*, but without achieving any actual reduction of femoral bone mineral density *in vivo*. A further assessment of bone turnover markers could be important to better elucidate the effect of conditioning on bone metabolism of MPS-I, in which the RANKL/OPG system is already altered^48^.

UCB represents a promising source of stem cells for early HSCT therapeutic approaches for several diseases that can be diagnosed at birth and has several advantages such as easy and quick procurement, absence of risk to donors, immediate availability, low risk of transmitting infections, greater tolerance of HLA disparity, and lower incidence of severe GVHD^1, 2, 4^. Furthermore, UCB has unique composition and biological characteristics, due to the presence of HSCs as well as a mixture of multipotent stem cells such as unrestricted somatic stem cells, mesenchymal stem cells, and endothelial colony-forming cells able to regenerate numerous tissue types with functional improvements^3, 49^. For example, the administration of human UCB cells into MPS-III B mice decreased behavioral abnormalities and tissue pathology^50^. In particular, the neuroprotective effect of human UCBCs seems to be a function of enzyme delivery and anti-inflammatory effect mediated by donor cells found throughout the brain and can be enhanced by repeated administrations^51^. We do not know whether neonatal UCBT could be also effective at preventing or reverting brain pathology in MPS-I disease. Although not investigated in our work, it is an important outstanding question that should be addressed in further studies.

Of note, UCB offers an alternative source of HSCs for gene therapy approaches, considering the possibility of collecting and storing autologous UCB at birth and reinfusing HSCs in the affected children after gene correction procedure. UCB-derived HSCs represent a particularly favorable target for gene therapy, given the reported high gene transfer rates^52^. Moreover, a neonatal gene therapy approach could help to achieve supra-normal enzyme activity in transplanted mice before disease manifestation, even in the case of low levels of chimerism. Interestingly, a pioneering study published by Simonaro *et al*. showed evidence of transduction of haematopoietic neonatal blood stem cells derived from MPS-VI cats and long-term persistence of retrovirally transduced cells into adult recipients^53^. Further studies would be needed to identify the best preparatory regimen suitable for transplanting affected neonates or infants^54^.

In conclusion, we demonstrated in an MPS-I mouse model the advantage of combining two factors that may allow for a better outcome in MPS-I patients: (1) early timing of the transplant and (2) the use of UCB, which is considered at the moment the best HSC source for this disease. This study serves as a proof of concept to develop early UCB transplantation strategies for newborns affected by genetic disorders, as well as an investigational platform for novel cell and gene therapy approaches for the treatment of genetic disorders diagnosed in the neonatal period.

## Materials and Methods

### Animals

C57BL/6-CD45.2 and C57BL/6-CD45.1 mice were purchased from Charles River Laboratories (Calco, Italy). The MPS-I mouse model (*Idua* ^−/−^ mice, C57BL/6-CD45.2 background)^10^ was purchased from The Jackson Laboratory (Bar Harbor, ME); a breeding colony was established and maintained from heterozygous mating pairs, and genotyping was performed on tail clips or ear snips DNA, as previously described^10^. Procedures involving animal handling and care conformed to institutional guidelines, in compliance with national laws and policies. Animals were used in accordance with a protocol approved by the Italian Ministry of Health (permit number 451/2015-PR).

### UCB and BM cells collection

UCBCs were collected at E18 of pregnancy from C57BL/6 pregnant dams, as previously described^24^. Briefly, pregnant females were euthanized by CO_2_ inhalation, the uterus was removed and cooled in ice-cold phosphate-buffered saline (PBS, Gibco). The fetuses were isolated, and the visceral yolk sac and amnion were removed. Then, the umbilical cord was cut, fetuses were transferred into warm heparinized PBS (10 U/mL), and blood was allowed to flow out. Fetuses were then euthanized.

BMCs were collected from the long bones of 6 to 10-weeks-old C57BL/6 mice by flushing.

UCBCs and BMCs were centrifuged at 670 g for 5 minutes, lysed using ACK (Ammonium-Chloride-Potassium) lysing buffer (StemCell Technologies) and counted in Bürker chamber with Turk solution.

### Transplantation procedures

#### Adult transplantation

For transplantation in adult mice, 6 to 8-weeks-old C57BL/6-CD45.1 females were conditioned by lethal irradiation, administered in two doses of 4.25 Gy (tot 8.5 Gy), using the X-ray irradiator Radgil (Gilardoni S.p.A.). CD45.2^+^ BMCs or UCBCs were transplanted by a single intravenous injection within 24 hours from conditioning.

#### Secondary mice

For secondary transplantation experiments, BMCs were harvested from primary mice at 4 months after transplantation and stained with anti-mouse CD45.2 PE antibody (clone 104, eBioscience). CD45.2^+^ BMCs were sorted using a FACSAria cell sorter (BD Biosciences) and re-transplanted into congenic C57BL/6-CD45.1 recipients (3 × 10^6^ cells/mouse) as described above.

#### Neonatal transplantation

UCBT in newborn mice was performed adjusting the scheme established by our group for the neonatal transplantation of murine BM^28^. 1 to 3-day-old pups from the MPS-I colony (CD45.2) were conditioned with a single intraperitoneal injection of 20 mg/kg busulfan (Busilvex, Pierre Fabre). 24 hours later, pups were transplanted by temporal vein injection of CD45.1^+^ healthy donor-derived UCBCs (2 × 10^5^ cells/mouse).

### FACS analysis

For flow cytometry analysis of haematopoietic subpopulations, UCBCs and BMCs were collected and processed as specified above. For engraftment evaluation in transplanted mice, 50 µL of peripheral blood (PB) were collected in heparin by tail bleeding and lysed with ACK buffer. BM was collected by flushing long bones, while splenocytes and thymocytes were collected by smashing the respective organ on a 70 µm cell strainer (Greiner Bio-One). Employed antibodies are listed in Supplementary Methods. Acquisition was performed on the FACSCanto™ II flow cytometer and the results were analyzed by FACS Diva software (BD Biosciences).

The levels of donor cell engraftment have been evaluated as: [donor CD45.1^+^/(donor CD45.1^+^  + recipient CD45.2^+^) × 100], (or the opposite in the case of the transplant of CD45.1^+^ mice with haematopoietic cells from CD45.2^+^ donor).

### Colony forming cell (CFC) assay

The CFC assay was performed in semi-solid medium supplemented with haematopoietic cytokines. Briefly, BMCs or UCBCs were resuspended in MethoCult GF M3434 (StemCell Technologies), plated in 35 mm low-adherence plastic dishes (Nunc) (20.000 cells/dish), and incubated at 37 °C and 5% CO_2_. Haematopoietic colonies were identified at day +14 by morphological observation on an inverted microscope. The nature of individual colonies was confirmed by picking them, cytospinning the cells on glass slides, and staining with May-Grünwald Giemsa.

### IDUA activity assay

IDUA activity was measured fluorimetrically in organs at 20 weeks after transplantation, as previously described^28, 55^. See the Supplementary Methods for details.

### Glycosaminoglycans quantification

GAGs were quantified in plasma and organs as described^28, 56^. See the Supplementary Methods for details.

### Bone phenotyping

Radiographic images of each limb and cranium were obtained by Faxitron MX-20 Specimen Radiography System (Faxitron X-ray Corp.) at an energy of 30 kV for 90 seconds with Eastman X-OMAT TL film (Eastman Kodak Co.) and processed by an automated X-ray film developer (Model M35A, Eastman Kodak Co.). Bone thickness was measured between the outer edges of cortical bone at the mid-diaphysis using ImageJ software (free from the NIH website).

Femurs from 20-weeks-old mice (n = 3 male mice/group) were scanned, using a SkyScan 1172 System (Bruker) with a source voltage and current of 65 kV and 153 μA, respectively. Following scanning, three-dimensional microstructural images were reconstructed using SkyScanNRecon software. SkyScan CT Analyzer software was used to calculate cortical micro-architectural parameters: cortical thickness (Ct.Th/mm), Total Area (TA/mm2), Bone Area (BA/mm2), and Medullary Area (MA/mm2).

### Histopathology

For the evaluation of bone morphology, hind limbs were fixed in 4% formaldehyde in phosphate buffer, decalcified in 10% EDTA (Sigma-Aldrich), routinely processed for paraffin embedding and used for qualitative and quantitative histological analysis. Four μm sections were deparaffinized, rehydrated, and stained with haematoxylin and eosin by standard procedures. For quantitative analysis, a semiautomatic image analyzer (IAS 2000, Delta System, Rome, Italy) was used to calculate the cortical thickness at the mid-diaphysis of the right femur and the area of the osteocytic lacuna within the same region of male WT, MPS-I, and MPS-I nUCBThi mice (n = 3 for each group, at least 100 osteocytic lacunae/mouse).

### *In vitro* osteoclasts differentiation

For osteoclastogenesis, mononuclear cells were isolated from the BM of 20-weeks-old mice as described above. Cells were plated at a density of 4 × 10^5^ cells per well in 96 multiwell plates in the presence of 1 μM dexamethasone (Sigma-Aldrich), 5 ng/ml TGF-β1 (RandD Systems), 25 ng/ml M-CSF (Peprotech), and 100 ng/ml RANKL (Peprotech), and cultured for 10 days. Medium and factors were replaced every 3 days.

Osteoclasts were detected 14 days after plating by cytochemical staining for TRAP (tartrate resistant acid phosphatase), using Leukocyte TRAP Kit 387-A (Sigma-Aldrich) according to the manufacturer’s instructions.

To determine the resorptive activity, cells were plated onto dentin discs (IDS) that were stained 21 days later by toluidine blue to quantify the resorbed area.

### Statistical analysis

All statistical analyses were performed using R freeware software. Continuous variables were contrasted between groups by the nonparametric Wilcoxon test for equality of the medians. All tests were two-sided with a 5% significance level, except those on the micro-CT data, area of osteocytic lacunae, osteoclast number, and percentage of resorbed area where a 1-sided alternative was considered. Data on the distribution of haematopoietic colonies subtypes were analyzed using Chi-square test. An ordinary regression model with binary regressors was applied to assess the impact of both disease and treatment. This enabled to separate the adverse effect of busulfan treatment from the therapeutic effect of UCBT on MPS-I.

## Electronic supplementary material

Supplementary Information
