# Supplementary material for: Neonatal umbilical cord blood transplantation halts skeletal disease progression in the murine model of MPS-I
Source: Sci Rep. 2017 Aug 25;7:9473. doi: 10.1038/s41598-017-09958-9 (PMC5573317; doi:10.1038/s41598-017-09958-9)
Supplement: Supplementary file 1 — Supplementary Information [file 41598_2017_9958_MOESM1_ESM.doc]

Neonatal umbilical cord blood transplantation halts skeletal disease progression in the murine model of MPS-I.

Isabella Azario^a^, Alice Pievani^a^, Federica Del Priore^a^, Laura Antolini^b^, Ludovica Santi^a^, Alessandro Corsi^c^, Lucia Cardinale^a^, Kazuki Sawamoto^d^, Francyne Kubaski^d,e^, Bernhard Gentner^f^, Maria Ester Bernardo^f^, Maria Grazia Valsecchi^b^, Mara Riminucci^c^, Shunji Tomatsu^d^, Alessandro Aiuti^f,g^, Andrea Biondi^h^, Marta Serafini ^a^*

**Supplementary Methods**

**Antibodies**

The following antibodies (eBioscience) were employed for flow cytometry analyses: anti-mouse CD45.1 PE (clone A20), anti-mouse CD45.1 APC (clone A20), anti-mouse CD45.2 PE (clone 104), anti-mouse CD45.2 APC (clone 104), anti-mouse CD45.2 PerCP-Cy5.5 (clone 104), anti-mouse CD3e PE (clone 145-2C11), anti-mouse CD45R (B220) PE (clone RA3-6B2), anti-mouse CD11b (Mac-1) PE (cloneM1/70), anti-mouse Ly-6G (Gr-1) FITC (clone RB6-8C5), anti-mouse TER-119 PE (clone TER-119), anti-mouse Ly-6A/E (Sca-1) APC (clone D7), anti-mouse CD117 (c-Kit) PE (clone 2B8), and anti-mouse Haematopoietic Lineage eFluor 450 cocktail.

**IDUA activity assay**

Organs (spleen, liver, heart, lungs, and kidneys) were harvested at sacrifice (20 weeks), frozen on dry ice and stored at -80°C. Portions of each organ were thawed and homogenized in 500 µL of 0.9% NaCl containing 0.2% Triton X-100 (Sigma-Aldrich) and a protease inhibitor cocktail (Sigma-Aldrich). The amount of protein in clarified supernatants of tissue homogenates was determined by Pierce BCA assay (Thermo Scientific). IDUA activity was then measured using the fluorogenic substrate 4-methylumbelliferyl-alpha-L-iduronide (Glycosynth). 5 µg of protein were added to a solution of 0.1 M sodium formate buffer, pH 3.2, containing 8 mM D-Saccharic acid 1,4-lactone and 0.4 mM 4-methylumbelliferyl-alpha-L-iduronide. Samples were incubated at 37°C for 1 hour, then the reaction was stopped by the addiction of 1 mL of 0.5 M carbonate buffer, pH 10.7. The fluorescence of the reaction product in the mix was read at 365 nm excitation and 488 nm emission wavelengths using a Tecan GENios microplate reader fluorometer (Tecan).

**Glycosaminoglycans quantification in tissues**

Portions of each organ collected at sacrifice were incubated overnight at 65°C with papain (Sigma-Aldrich), and then clarified for 10 min at 9391 g. GAG levels were measured using the Blyscan Sulfated Glycosaminoglycan colorimetric assay (Biocolor) according to the manufacturer’s instructions. Chondroitin 4-sulfate was used as standard. Samples were read at 620 nm emission wavelength using a Tecan GENios microplate reader fluorometer (Tecan), and GAG levels were expressed as μg GAGs/mg protein in each sample.

**Glycosaminoglycans quantification in plasma**

At sacrifice, peripheral blood was collected in EDTA, and plasma was obtained by centrifugation at 587 g for 10 min and stored at -80°C. Ten μl of each plasma sample and 90 μl of 50 mM Tris–hydrochloric acid buffer (pH 7.0) were placed in wells of AcroPrep™ Advance 96-Well Filter Plates (OMEGA 10K, PALL Co). The filter plates were placed on the receiver and centrifuged at 2000 g for 15 min to remove free disaccharides. The membrane plates were transferred to a fresh receiver plate. Ten μl of IS solution (5 μg/ml), 20 μL of 50 mM Tris-HCl buffer, and 10 μL of chondroitinase B, heparitinase, and keratanase II (each 2 mU/10 μL of 50 mM Tris-HCl buffer) were added onto each filter. The plate was incubated at 37°C for 5 hr and centrifuged at 2000 g for 15 min. The receiver plate containing disaccharides was stored at -20°C until injection to liquid chromatography tandem mass spectrometry (LC-MS/MS).

The chromatographic system consisted of 1260 Infinity (Agilent Technologies) and Hypercarb column (2.0 mm i.d. 50 mm, 5 µm, Thermo Electron). The mobile phase was a gradient elution from 0.025% ammonia to 90% acetonitrile in 0.025% ammonia. The 6460 Triple Quad mass spectrometer (Agilent Technologies) was operated in the negative ion detection mode with thermal gradient focusing electrospray ionization (Agilent Technologies). Specific precursor ion and product ion were used to detect and quantify each disaccharide. A m/z 354.29 precursor ion and m/z 193.1 product ion was used to detect the IS (chondrosine). Peak areas for all components were integrated automatically using QQQ Quantitative Analysis software (Agilent Technologies). The concentration of each disaccharide was calculated using QQQ Quantitative Analysis software.

**Supplementary Figures and Tables**

**Table S1. Frequency of the different haematopoietic subpopulations in UCB and BM.**

|  | **UCB** | **BM** | **P value** |
| --- | --- | --- | --- |
| **T cells**  **(CD3^+^ in CD45^+^)** | 2.5%  (range from 1.4% to 2.8%) | 3.6%  (range from 2.9% to 6.0%) | 0.0147 |
| **B cells**  **(B220^+^ in CD45^+^)** | 4.7%  (range from 3.6% to 8.7%) | 10.6%  (range from 7.9% to 13.2%) | 0.0571 |
| **Myeloid cells**  **(Mac-1^+^ in CD45^+^)** | 75.4%  (range from 73.1% to 77.7%) | 79.5%  (range from 77.8% to 81.0%) | 0.0286 |
| **Myeloid cells**  **(Gr-1^+^ in CD45^+^)** | 54.3%  (range from 48.6% to 56.8%) | 76.0%  (range from 71.6% to 78.5%) | 0.0143 |
| **Erythrocytes**  **(Ter119^+^)** | 67.4%  (range from 61.8% to 78.9%) | 2.9%  (range from 0.7% to 4.1%) | 0.0500 |

The median percentages of the different haematopoietic subpopulations in UCB and BM are reported (n≥3 for UCB, n≥3 for BM). P values calculated by 1-sided Wilcoxon test.

**Table S2. Effect of conditioning on bone measurements.**

|  | Variables | | |
| --- | --- | --- | --- |
|  | β1 coeff (p value) | β2 coeff (p value) | γ coeff (p value) |
| **Skull width** | 4.39 (0.0003) | -2.66 (0.0151) | -3.76 (0.0012) |
| **Zygomous width** | 12.96 (<0.0001) | -1.23 (0.235) | -8.06 (<0.0001) |
| **Femur width** | 6.25 (<0.0001) | -1.27 (0.2194) | -2.83 (0.0105) |
| **Humerus width** | 6.96 (<0.0001) | -2.81 (0.0109) | -2.76 (0.0121) |

Anova regression model on categorical variables. β1 is the effect on the parameter considered due to disease; β2 is the nUCBT effect on WT; γ is the differential effect of nUCBT on MPS I.

**Figure S1. Multi-lineage UCB-derived reconstitution within BM, spleen, and thymus of a recipient mouse at 6 months after nUCBT**

**
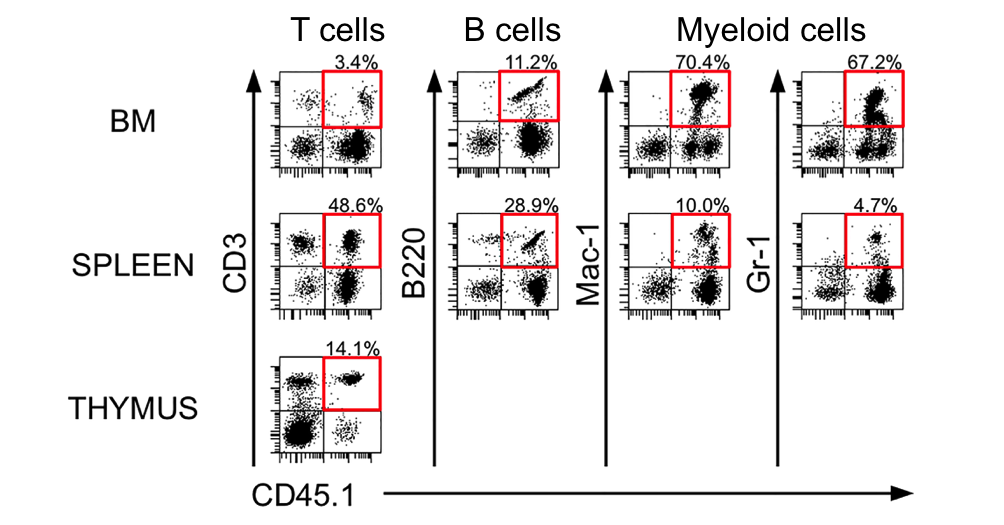
**

Representative lineage distribution of UCB-derived cells in the BM, spleen, and thymus of recipient mice at 6 months after nUCBT. Dot plots to determine donor-derived T cells (CD45.1^+^CD3^+^), B cells (CD45.1^+^B220^+^), and myeloid cells (CD45.1^+^Mac-1^+^ and CD45.1^+^Gr-1^+^) are shown. Percentages in total leukocytes are indicated.

**Figure S2. Morphology of fetal red blood cells**

**
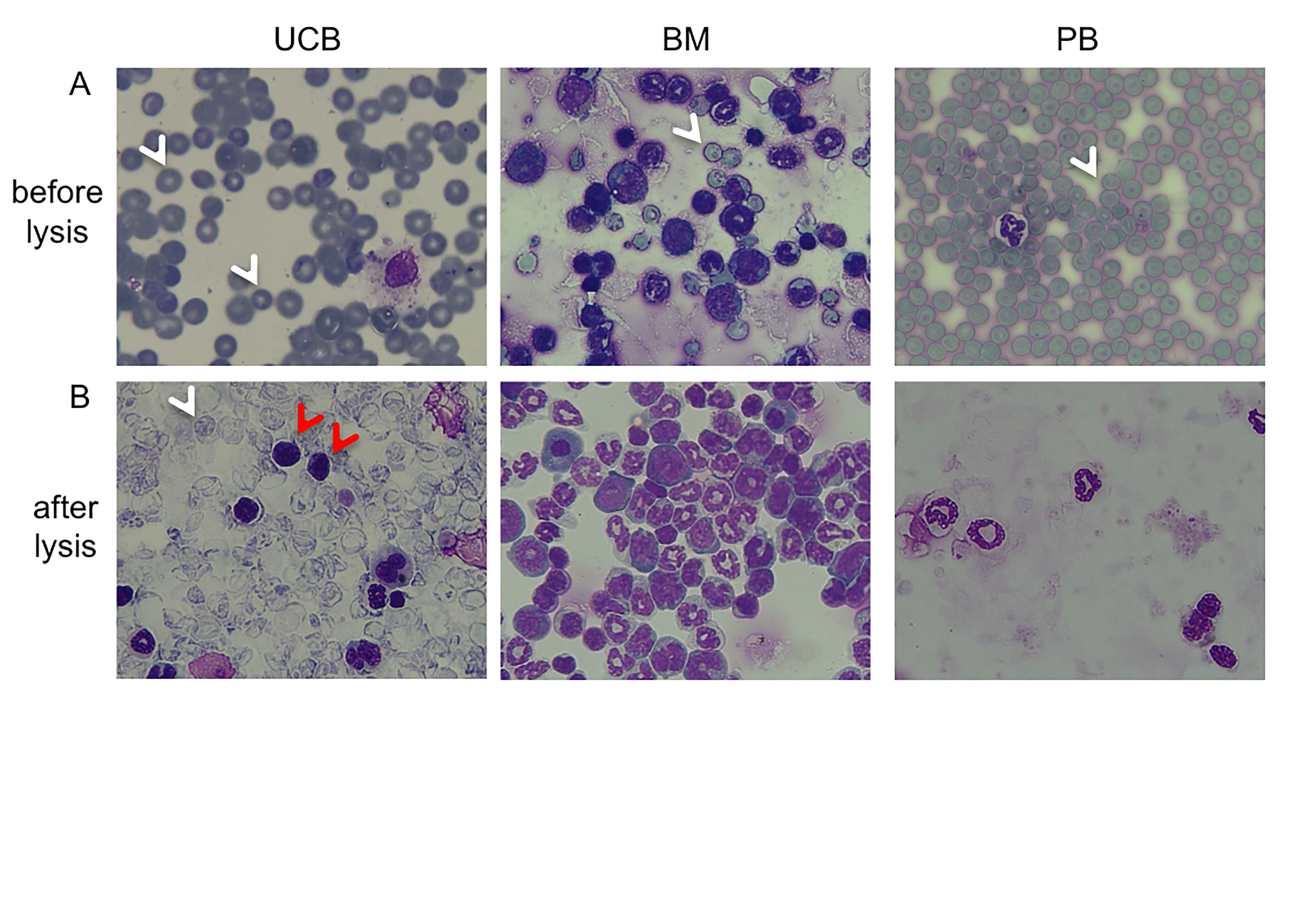
**

A) Smears of day 18-fetus UCB, adult BM, and PB. Red blood cells were indicated by white arrows. Note the heterogeneity in size and color of fetal red blood cells (Magnification 50x, May Grunwald-giemsa staining). B) Cytospin preparations of the same samples after lysis. Red blood cells resistant to hypotonic lysis (white arrow) and nucleated erithroblasts (red arrows) can be found in the umbelical cord blood sample (Magnification 50x, May Grunwald-giemsa staining).

**Figure S3. Bone mineral density of the femurs of WT, MPS-I, and MPS-I nUCBT-hi mice.**


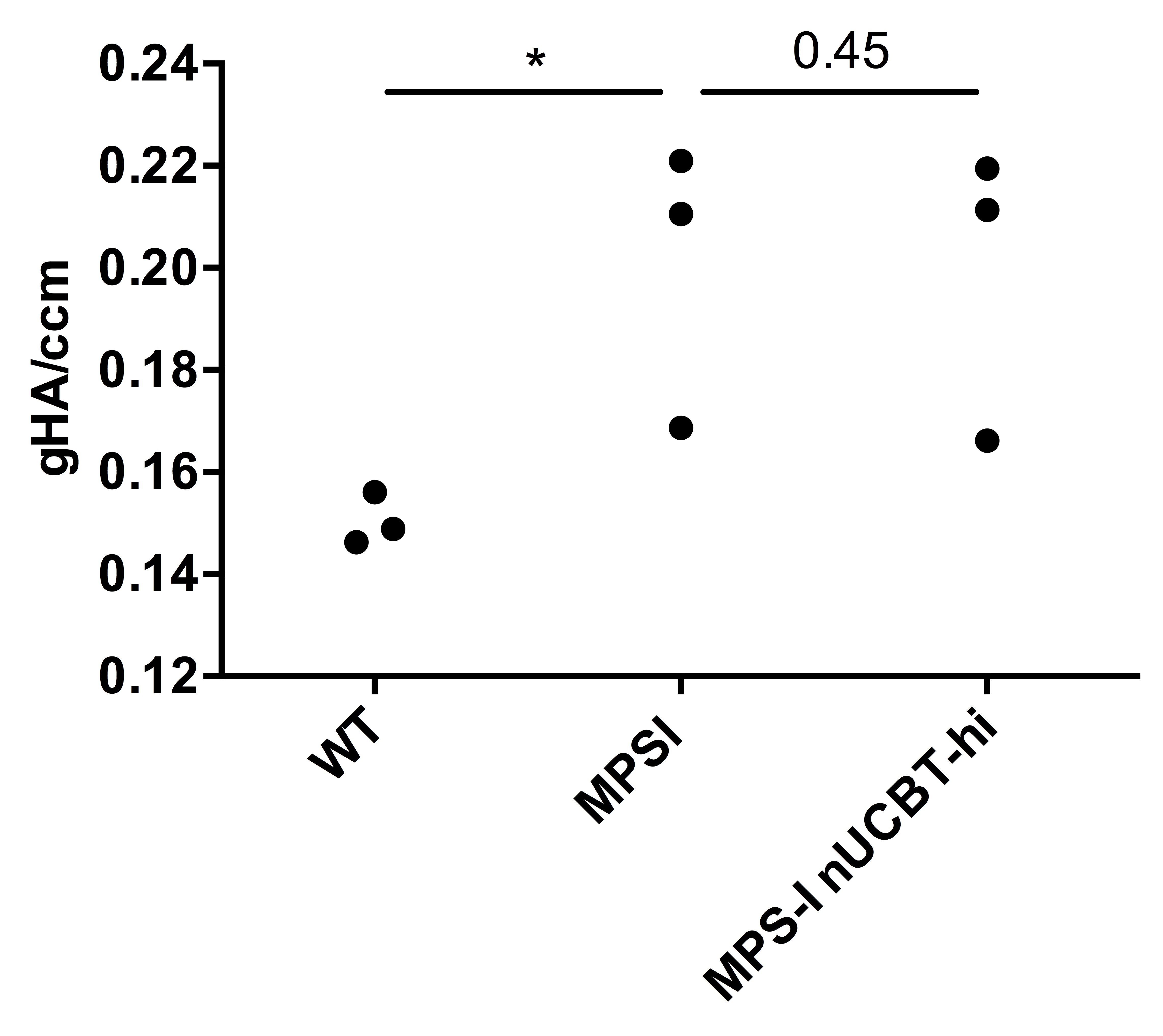


The graph represents the measurement of bone mineral density evaluated in the femurs of 20 weeks old WT, MPS-I, and MPS-I nUCBT-hi mice (n=3 for each group).
